# Reply to Wüster et al. On the Importance of Correct Snake Identification. Comment on “Chippaux et al. Snakebites in Cameroon by Species Whose Effects Are Poorly Described. *Trop. Med. Infect. Dis.* 2024, *9*, 300”

**DOI:** 10.3390/tropicalmed11010032

**Published:** 2026-01-22

**Authors:** Jean-Philippe Chippaux, Fabien Taieb

**Affiliations:** 1 MERIT Unit, Institut de Recherche pour le Développement, Paris Cité University, F-75006 Paris, France; 2 Institut Pasteur Medical Center, Paris Cité University, F-75015 Paris, France

As Wüster et al. [1] point out, identifying the snakes responsible for bites is crucial information for improving our understanding of the epidemiology of snakebites and the clinical symptoms of each venomous species. However, snake identification is a complex process that only a few specialists can perform, and even they disagree when identifying specimens, especially from poor-quality photos. Furthermore, since photographers are usually neither herpetologists nor naturalists, their photos often fail to capture the distinguishing features necessary for identification, which vary by species.

In fact, only about one-third of victims bring in the snake responsible for the bite, and identification is not usually performed at primary care facilities, which treat nearly all patients [2]. Therefore, clinical examination is the main guide for healthcare personnel, as biological tests are not always feasible. Antivenoms are usually polyvalent, providing coverage for as many venomous species as possible in the region where they are used.

We are grateful to Wüster et al. for drawing attention to this issue, enabling us to overcome long-standing challenges associated with identifying incomplete, damaged or poorly photographed specimens.

Wüster et al. reported errors in identifying some of the specimens responsible for envenomation, which are shown in our article [3].

The first issue relates to the Naja species shown in Figure 7 of the article [3]. The bite occurred in Tokombéré, a town located in Cameroon’s Far North Region close to the Nigerian border. We attributed this to *N. haje*, but they believe it to be *N. nigricollis*.

As one of the authors of this commentary rightly points out, “*N. haje* can usually be distinguished from other African cobras by examination of the headscales (Broadley, 1968) [4]. The eye is usually separated from the upper lip scales by a row of small scales, but in some specimens, including the snake which bit Case 1, the third upper lip scale may enter the orbit causing confusion with the forest cobra (*N. melanoleuca*) and *N. nigricollis* (Anderson, 1898)” [4]. The specimen shown in Figure 7 [3] does not have any upper labial plates in contact with the eye, which is characteristic of *N. haje*. To our knowledge, one upper labial always enters the eye in *N. nigricollis*. Regardless of how the specimen in Figure 7 [3] is identified, abnormalities can be seen in the scales surrounding the eye. According to Roman, in *N. haje*, “the eye is separated from the labials by suboculars that are sometimes very small in 38 specimens; in one specimen, a labial on each side touches the eye; and in two other specimens, the labial touches the eye on only one side. There is one preocular in 29 specimens, two on one side of the head in six specimens and two preoculars in the remaining three specimens” [5]. In other words, out of 41 *N. haje* specimens, Roman identified 9–12 with an anomaly in the scales bordering the eye, accounting for at least 22% of the total. However, Roman found no such abnormalities in the scales bordering the eye, nor any subocular scales on either side of the head, in 100 *N. nigricollis* specimens. Additionally, two subocular scales were present without any contact with the anterior temporal scales, distinguishing them from postoculars. Therefore, it is more likely that the pre-ocular scale fuses with a subocular scale in *N. haje* than that two subocular scales are present in *N. nigricollis*. Furthermore, *N. haje* has one anterior temporal scale, like our specimen, whereas *N. nigricollis* has two [6]. It is possible that the large scale under the anterior temporal is not an upper labial, as we thought, but a second anterior temporal. However, the fact that it is difficult to count the upper labials, and, in particular, to visualize the fourth and fifth ones, makes it impossible to settle the question. Table 1 summarizes the data from the cephalic scales. We have therefore identified this specimen as *N. haje*.

We agree that the clinical presentation is more consistent with envenomation by *N. nigricollis*. However, this does not rule out *N. haje* being the cause. As Warrell et al. reported [4], envenomation by *N. haje* can present as cytotoxic envenomation. Neurotoxic effects are often present but may be absent. For instance, Wüster et al. did not consider *N. melanoleuca* bites in our study: of the five cases of envenomation, three patients presented symptoms of cytotoxicity without neurotoxic signs. As *N. melanoleuca* bites are more frequent than *N. haje* bites, this could explain why this type of exception has been noted for *N. melanoleuca* envenomation, but not for *N. haje* envenomation.

Wüster et al. then refute the identification of the specimen in Figure 8 [3], which they attribute to *N. nigricollis* rather than *N. katiensis*. The bite also occurred in Tokombéré.

The coloration of *N. nigricollis* differs from that of *N. katiensis* [5,6]. In *N. nigricollis*, the belly is dark with more or less light transverse bands and the first ventral scales are black, which is not the case with our specimen. In contrast, the belly of *N. katiensis* is light with one or two dark bands at the neck, and the first ventral scales are light. In addition, the dorsal scales “have a dark border near the edge” [5]. Photos of the belly and back of the same specimen reveal that (a) the belly is entirely light in color (Figure 1), except for the dark band on the neck shown in the photo in the article, and (b) the dorsal scales are edged with a dark border (Figure 2).

Finally, Wüster et al. dispute the identification of the three specimens shown in Figure 11 [3].

Figure 11A [3] shows *D. jamesoni*, not *D. viridis*. This is not an identification error, but rather a slip of the pen. We never mentioned *D. viridis* elsewhere in this article, as it is absent from Cameroon, but we always referred to *D. jamesoni*. Unfortunately, we did not spot this error, and neither did the reviewers.

Figure 11B [3] shows a species of the genus *Causus*. This identification is possible, though not certain, given the poor condition of the specimen and its worn skin.

We agree that the specimen in Figure 11C [3] is *Bitis arietans*. We apologize for this gross misidentification.

We acknowledge—and apologize for—one misidentification (Figure 11C [3]). The identifications by Wüster et al. in Figures 7, 8, and 11B [3] are disputable. The identification in Figure 11A [3] was a slip of the pen.

However, it is incorrect to extrapolate the error rate of the 12 snakes shown in photos in the paper to the 151 snakes identified in this study. This study is ancillary to a clinical study that aimed to evaluate the efficacy and tolerability of an antivenom under real-life conditions. Identifying the snakes responsible for the bites was not the initial intention. It was also not planned to preserve the specimens. In most cases, identification is possible based on photographs. However, identifying the snake may be uncertain or controversial in a few circumstances when only poor-quality photos are available.

In general, snakebites in sub-Saharan Africa have a significant impact on morbidity and mortality. Healthcare workers in decentralized areas are on the front line. Progress in developing appropriate management strategies in this context has been modest in recent years. Therefore, open dialogue and patient-centered solutions should be prioritized to effectively address this issue.

## Figures and Tables

**Figure 1:**
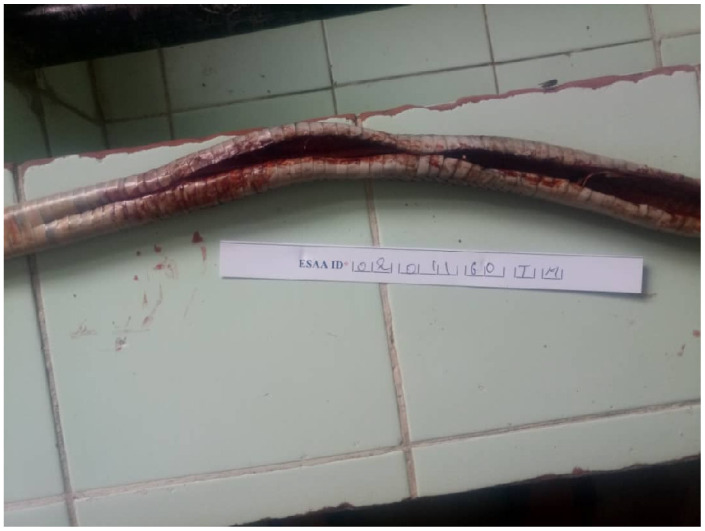
Ventral side of the specimen.

**Figure 2:**
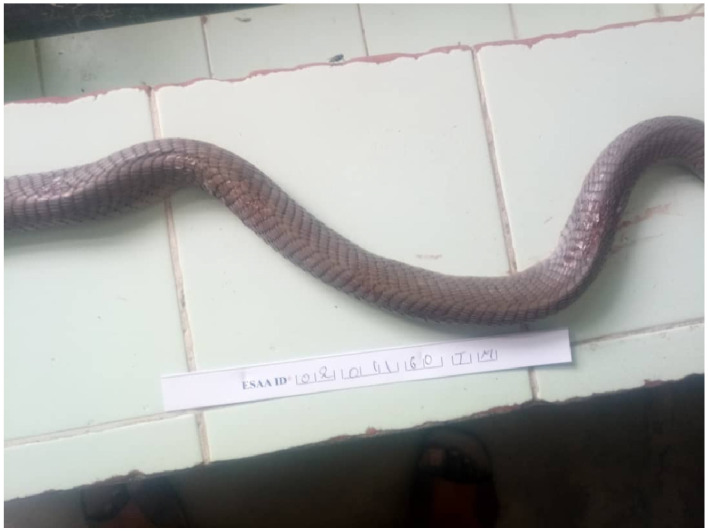
Dorsal side of the specimen.

**Table 1:** Summary of the characteristics of the cephalic scales of *N. haje*, *N. nigricollis*, and *N. melanoleuca*.

Scales	*N. haje*	*N. nigricollis*	*N. melanoleuca*	This Specimen
Preoculars	1, sometimes 2	1 or 2, most often 2	1	2
Upper labials entering eye	0	1 (3^rd^)	2 (3^rd^ and 4^th^)	0
Suboculars	2	1	1	2
Postoculars	2 or 3	2 or 3	2 or 3	2
Temporals (1st row)	1	2	1	1
Frontal	As long as wide	Longer than wide	As long as wide	As long as wide
